# Molecular basis of telaprevir resistance due to V36 and T54 mutations in the NS3-4A protease of the hepatitis C virus

**DOI:** 10.1186/gb-2008-9-1-r16

**Published:** 2008-01-23

**Authors:** Christoph Welsch, Francisco S Domingues, Simone Susser, Iris Antes, Christoph Hartmann, Gabriele Mayr, Andreas Schlicker, Christoph Sarrazin, Mario Albrecht, Stefan Zeuzem, Thomas Lengauer

**Affiliations:** 1 Department of Computational Biology and Applied Algorithmics, Max Planck Institute for Informatics, 66123 Saarbrücken, Germany; 2 Department of Internal Medicine I, Johann Wolfgang Goethe University Hospital, 60590 Frankfurt/Main, Germany; 3 Department of Internal Medicine II, Saarland University Hospital, 66421 Homburg/Saar, Germany

## Abstract

**Background:**

The inhibitor telaprevir (VX-950) of the hepatitis C virus (HCV) protease NS3-4A has been tested in a recent phase 1b clinical trial in patients infected with HCV genotype 1. This trial revealed residue mutations that confer varying degrees of drug resistance. In particular, two protease positions with the mutations V36A/G/L/M and T54A/S were associated with low to medium levels of drug resistance during viral breakthrough, together with only an intermediate reduction of viral replication fitness. These mutations are located in the protein interior and far away from the ligand binding pocket.

**Results:**

Based on the available experimental structures of NS3-4A, we analyze the binding mode of different ligands. We also investigate the binding mode of VX-950 by protein-ligand docking. A network of non-covalent interactions between amino acids of the protease structure and the interacting ligands is analyzed to discover possible mechanisms of drug resistance. We describe the potential impact of V36 and T54 mutants on the side chain and backbone conformations and on the non-covalent residue interactions. We propose possible explanations for their effects on the antiviral efficacy of drugs and viral fitness. Molecular dynamics simulations of T54A/S mutants and rotamer analysis of V36A/G/L/M side chains support our interpretations. Experimental data using an HCV V36G replicon assay corroborate our findings.

**Conclusion:**

T54 mutants are expected to interfere with the catalytic triad and with the ligand binding site of the protease. Thus, the T54 mutants are assumed to affect the viral replication efficacy to a larger degree than V36 mutants. Mutations at V36 and/or T54 result in impaired interaction of the protease residues with the VX-950 cyclopropyl group, which explains the development of viral breakthrough variants.

## Abstract 2

Structural analysis of the inhibitor Telaprevir (VX-950) of the hepatitis C virus (HCV) protease NS3-4A shows that mutations at V36 and/or T54 result in impaired interaction with VX-950, explaining the development of viral breakthrough variants.

## Background

More than 170 million people worldwide are chronically infected with the hepatitis C virus (HCV). Combination therapy with pegylated interferon-α plus ribavirin shows sustained virologic response rates of approximately 50% in HCV genotype 1 infected patients [1-3], which emphasizes the need for new antiviral drugs. The serine protease NS3-4A is a promising drug target for specific antiviral treatment. HCV genotypes exhibit about 80% sequence identity in NS3-4A, with highly conserved key residues [4]. NS3-4A is bifunctional, possessing a protease as well as a helicase domain. Especially the protease domain is a target for rational drug design [5-8]. The serine protease has a chymotrypsin fold, which consists of the amino-terminal 181 amino acids of NS3. The three catalytic residues H57, D81 and S139 are located in a crevice between the two protease β-barrels [9-11]. The numbering used in the following is according to the structure 1DY8[12] taken from the Protein Data Bank (PDB) [13,14]. The central region of NS4A is buried almost completely inside NS3 and serves as a cofactor for proper folding of NS3 [9].

The binding pocket of the protease is shallow, non-polar, and rather difficult to target. Therefore, the development of potent protease inhibitors has been a challenging task in the past. This is reflected by the variety of rational drug design approaches and drug candidates tested so far, for example, protease substrate or product analogs, serine-trap inhibitors, tripeptide inhibitors and *de-novo *peptidomimetics [6,15]. Data for drug resistance and antiviral efficacy have been published for the protease inhibitors BILN-2061 (ciluprevir) [16,17], VX-950 (telaprevir) [18-20], and SCH 503034 (boceprevir) [21,22].

**Figure 1:**
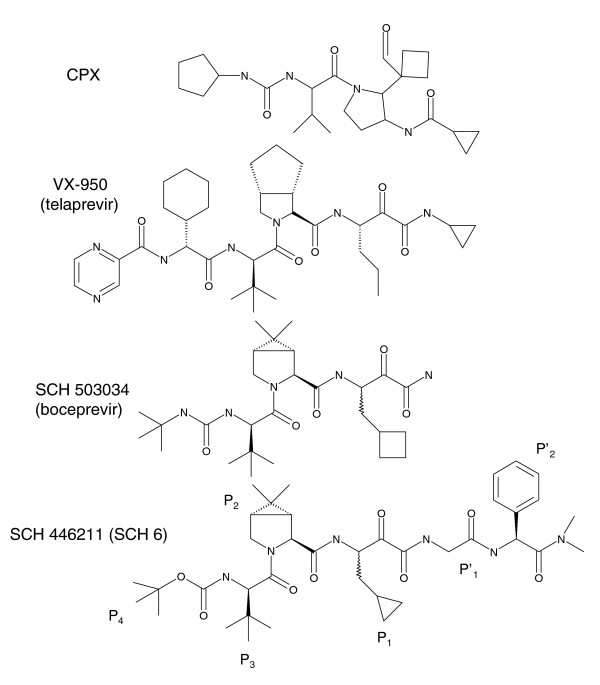
Molecular structures of the NS3-4A serine protease inhibitors VX-950 (telaprevir) and SCH 503034 (boceprevir) as well as of the co-crystallized protease ligands CPX and SCH 446211. The P_1 _to P_4 _and P'_1 _to P'_2 _groups are numbered according to the nomenclature of Schechter and Berger [61]. Residues surrounding a cleavage site are designated from the amino- to carboxyl-terminus, that is, P_4_-P_3_-P_2_-P_1 _P'_1_-P'_2_-P'_3_-P'_4_, with the scissile bond between P_1 _and P'_1_. They are annotated only for SCH 446211.

VX-950 is a tetrapeptidic compound with α-ketoamide as active-site binding motif, covalently bound to S139 [23-25]. Figure 1 shows the chemical structure of VX-950 in comparison with other ligands. Strong antiviral efficacy for VX-950 was demonstrated *in vivo *during a phase 1b clinical trial, with an HCV RNA decline above 3 log after treatment duration of only 24 hours [18]. As observed with other specific antiviral agents, the treatment efficacy diminished over time, due to the selection of drug-resistant viral variants. Mutations that confer drug resistance to VX-950 were detected independently in different patients within two weeks of treatment. They have been found at four different sites: V36, T54, R155 and A156 [18,19,26]. *In vitro *drug resistance was quantified by enzymatic, inhibitory concentration 50% (IC_50_) values [19,26-28]. Viral fitness and corresponding replication efficacies were measured by HCV RNA levels [19,26-28].

R155 and A156 are localized in the binding pocket of the protease NS3-4A. A156 interferes directly with protease inhibitor binding and leads to high-level drug resistance [19]. An extensive analysis of HCV quasispecies revealed single mutants at positions V36, T54 and R155, and double-mutants at V36/R155 in all breakthrough patients investigated [19]. V36, T54 and R155 mutants confer low- to medium-level drug resistance, and an inverse relationship between *in vivo *viral fitness and drug resistance was observed [19]. The mutations are associated with an intermediate reduction in viral replication efficacy. Mutations at position V36 conferred low-level resistance to VX-950 with a mean IC_50 _value of 226 nM and an IC_50 _range of 110 nM to 444 nM, compared with the HCV reference strain, genotype 1a. Interestingly, the T54S mutant was associated with low-level resistance and a mean IC_50 _value of 120 nM, while the T54A mutant showed a higher level of resistance with a mean IC_50 _value of 749 nM. *In vitro *IC_50 _data and corresponding IC_50 _fold changes in resistance over the HCV genotype 1a reference strain are summarized for VX-950 in Table 1[19,26,28]. Molecular mechanisms leading to drug resistance at R155 and A156 have been investigated [19,20], whereas the reason for drug resistance mutants at V36 and T54 is still unknown. The present work investigates the molecular basis for VX-950 resistance at V36 and T54.

**Table 1:** Enzymatic *in vitro *drug resistance data for telaprevir (VX-950)

	IC_50 _mean (nM)	IC_50 _range (nM)	IC_50 _(fold changes)
HCV genotype 1a	70	-	-
V36A/G/L/M	226	110-444	1.7-6.9
T54S	120	-	1.9
T54A	749	-	11.7
R155G/I/K/L/M/S/T	538	275-1,050	4.3-16.4
A156I/T/V	29,800	12,500-50,000	195-781
A156S	1,400	-	21.9

Enzymatic *in vitro *resistance data for VX-950 (mean IC_50 _values, IC_50 _range and IC_50 _fold changes) are compared to the reference strain HCV-H, genotype 1a (UniProtKB accession number P27958) and are shown for VX-950 inhibition of wild-type and mutant NS3-4A proteases (V36, T54, R155 and A156) [19,26,28].

## Results

The following sections describe the results of the analysis of the HCV protease structure of NS3-4A and the different ligand interaction modes using alternative experimental structure models. The ligand binding mode of the inhibitor VX-950 was investigated by computational protein-ligand docking. Structural changes in the binding pocket and the catalytic triad of the protease were characterized by molecular dynamics simulations of T54A/S mutants and rotamer analysis of V36A/G/L/M side chain conformations. A residue-based network of non-covalent interactions was constructed to investigate molecular mechanisms of drug resistance. Experimental data are provided for the V36G mutant to corroborate our findings. The last section comprises a sequence analysis of HCV genotypes and their polymorphisms with respect to the mutational sites discussed in this study.

### Analysis of NS3-4A protease structures and ligand binding modes

**Figure 2:**
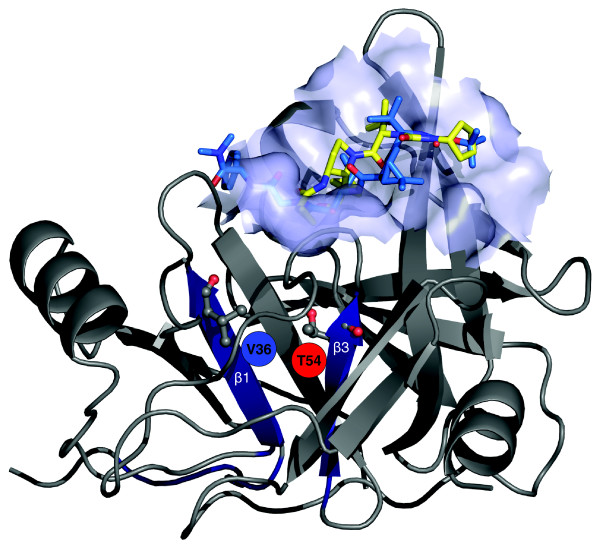
NS3-4A protease domain of PDB structure 1RTL with co-crystallized ligand CPX (yellow) [32] and a second ligand, SCH 446211 (light blue), taken from the superimposed PDB structure 2FM2 [30]. The protease binding pocket from structure 1RTL is shown as a transparent surface patch. The residues V36 and T54 are depicted as stick-and-ball models, located in the parallel β-strands β1 and β3 of an anti-parallel β-sheet (dark blue).

The mutated positions V36 and T54 are buried in the protease domain of NS3-4A in the two β-strands β1 and β3 of an anti-parallel β-sheet (Figure 2). T54 is at the very end of the strand β3, next to a loop directly involved in the ligand binding cavity at the protein surface. The side chains of V36 and T54 point towards each other. We identified a buried cavity between V36 and T54 and calculated the cavity size in the wild-type and in the T54A mutant. Comparison of the volumetric data for both cavities indicates no significant difference in size. Both mutated sites are located close to a hydrophobic cavity of the ligand binding pocket at the protein surface (Figure 2). Superposition of alternative experimental structures of NS3-4A was used to determine conformational changes of the protein structure and the binding modes of different co-crystallized protease ligands. The backbone is conserved in most parts. The three residues Q41, I132 and D168 near the ligand binding site show considerable variability in their side chain conformations. In addition, the catalytic residue H57 adopts a different side-chain conformation when the protease binds an inhibitor derived from 2-aza-bicyclo[2.2.1]heptane-3-carboxylic acid (PDB entry 2F9U[29]). We identified an experimental protease structure (PDB entry 2FM2[30]) containing the SCH 446211 ketoamide inhibitor (SCH 6), which is similar to VX-950. The ligand scaffolds of these two inhibitors differ only in the region of the scissile bond and the P'_1 _group (Figure 1). Therefore, a similar binding mode for VX-950 and SCH 446211 is expected [31]. In addition, the PDB entry 1RTL[32] includes a protease bound to the ligand CPX (N-[(2R,3S)-1-((2S)-2-{[(cyclopentylamino)carbonyl]amino}-3-methylbutanoyl)-2-(1-formyl-1-cyclobutyl)pyrrolidinyl]cyclopropanecarboxamide). CPX and the VX-950 compound include a cyclopropyl group at an equivalent position (Figure 1). The cyclopropyl group of the CPX ligand is tightly bound into a narrow hydrophobic cavity at the protease surface of 1RTL (Figure 2). Presumably, the cyclopropyl group of VX-950 is oriented towards the same hydrophobic cavity.

**Figure 3:**
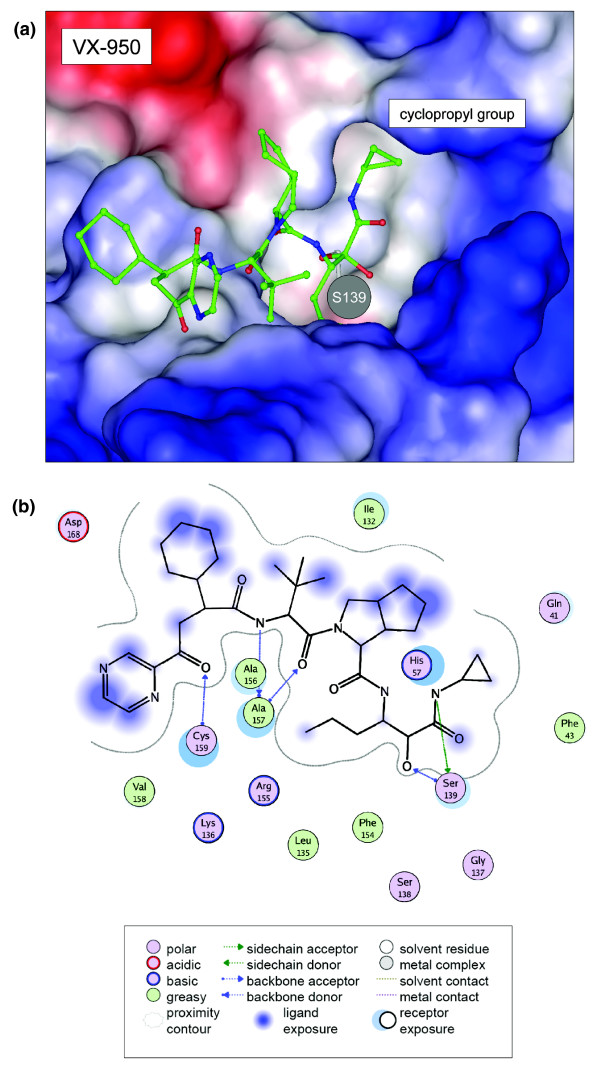
VX-950 protein-ligand binding. **(a) **Surface representation of the NS3-4A protease binding pocket (PDB entry 1RTL) with the docked VX-950 compound. VX-950 is covalently bound to S139. The cyclopropyl group is oriented towards a hydrophobic cavity. The surface of the protein was colored with the vacuum electrostatics function of PyMOL. Charges are computed with the Amber 99 force field and projected on the protein surface, whereas colored patches (red = positive, blue = negative) denote polar regions and white patches apolar protein regions. **(b) **MOE plot for interactions of the protease with the VX-950 compound. The legend is at the bottom.

We docked the compound VX-950 to the NS3-4A protease to determine its conformation in the ligand binding pocket (Figure 3). FlexX generated nine different placements of VX-950, and the top-ranking placement exhibits a binding mode comparable to that of the 2FM2 ligand SCH 446211. As expected from the structure analysis detailed above, the VX-950 cyclopropyl group is placed towards the hydrophobic cavity in the ligand binding pocket, similar to the placement of the cyclopropyl group of CPX in 1RTL. The cyclopropyl group is buried in the surface cavity and faces towards the aromatic ring of F43. The binding modes of the 1RTL and 2FM2 ligands CPX and SCH 446211, respectively, are given in Figure S1 in Additional data file 1.

**Figure 4:**
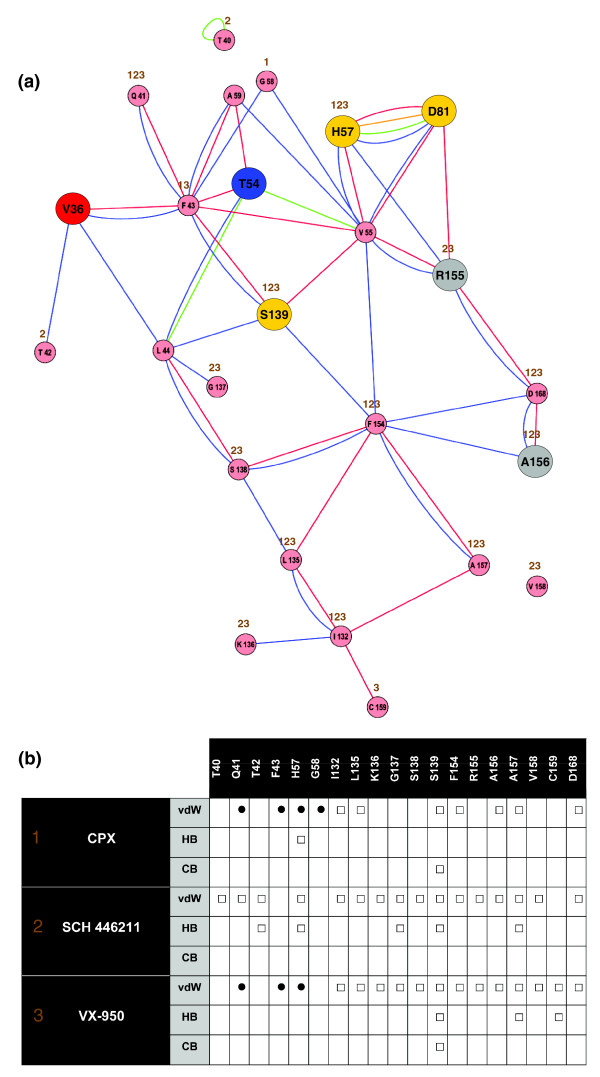
Network of non-covalent residue interactions for the NS3-4A protease and the corresponding list of protein-ligand interactions. **(a) **Network analysis of non-covalent residue interactions for the NS3-4A protease (PDB entry 1RTL). Nodes represent residues and colored edges represent different types of interactions: van der Waals interactions, backbone-side chain (blue), side chain-side chain (red); H-bond interactions, backbone-side chain (green), side chain-side chain (orange). Protein-ligand interactions for the 1RTL and 2FM2 ligands CPX and SCH 446211, respectively, as well as for VX-950 are tagged by brown Arabic numerals above each residue node (see (b)). Catalytic residues are yellow and the mutated residues are blue (V36), red (T54) and grey (R155, A156). **(b) **List of van der Waals interactions (vdW), H-bonds (HB) and covalent bonds (CB) for the 1RTL and 2FM2 ligands CPX and SCH 446211, respectively, and the VX-950 ligand docking result. Each dot or square represents one interaction of the ligand with an amino acid of the NS3-4A protease, and dots indicate interactions with the cyclopropyl group. Brown Arabic numerals refer to protein-ligand interactions in the network of non-covalent interactions (a).

A two-dimensional network of non-covalent, hydrogen bonds (H-bonds) and van der Waals, interactions between amino acids (Figure S2 in Additional data file 1) was generated based on the PDB structure model 1RTL of the protease NS3-4A. We selected a subset of the complete network, including the catalytic triad of the protease NS3-4A, the mutational sites V36, T54, R155 and A156, and other residues involved in interactions with VX-950, CPX and SCH 446211 (Figure 4). The ligand CPX forms interactions with the cyclopropyl group by van der Waals interactions at Q41, F43, H57 and G58 (Figure 4), but the ligand SCH 446211 interacts only with Q41 and H57, but not F43 and G58. No interaction can be observed with the mutational sites V36 and T54 in the case of the ligand CPX and SCH 446211 (Figure 4). The docking result for VX-950 predicts van der Waals interactions of the cyclopropyl group with Q41, F43 and H57. Protein-ligand interactions for the ligands CPX and SCH 446211 as well as for VX-950 docking are summarized in the list included in Figure 4.

### Mutations at position T54

**Figure 5:**
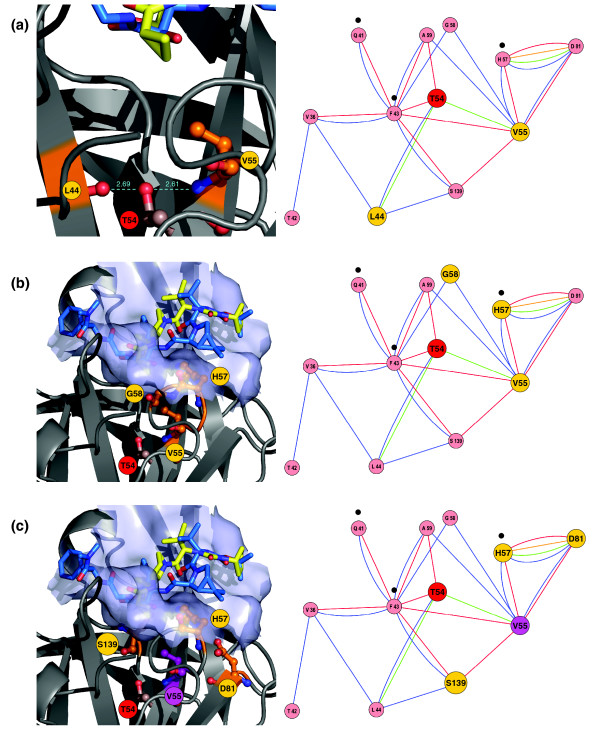
Structure and network analysis of non-covalent residue interactions for T54. Left column: visualization of the NS3-4A protease structure and surface of the binding pocket of 1RTL with co-crystallized ligands taken from two superimposed PDB structures: 1RTL with ligand CPX (yellow) and 2FM2 with ligand SCH 446211 (light blue). Right column: corresponding network analysis of non-covalent residue interactions for T54 mutants. Residues presumed to interact with the cyclopropyl group of VX-950 are indicated by black dots. Nodes represent residues and colored edges represent different types of interactions (see Figure 4): van der Waals interactions, backbone-side chain (blue), side chain-side chain (red); H-bond interactions, backbone-side chain (green), side chain-side chain (orange). **(a) **Anti-parallel β-sheet and H-bond interactions of T54 with L44 and V55 (yellow). H-bonds are shown as cyan dotted lines and corresponding distances printed in cyan. **(b) **Loop-forming residues (orange) and hydrophobic pocket conformation. **(c) **Impact of T54 mutants on the catalytic triad via the node V55 (purple).

T54 is located at the very end of the β-strand β3 (Figure 2), which belongs to an anti-parallel β-sheet. The hydroxyl group of the T54 side chain is involved in the formation of two H-bonds with residues V55 and L44 in the strands β3 and β1, respectively (Figure 5a). In the wild-type structure, the tip of the β3-strand turns slightly away from the neighboring β1-strand (Figure 5a) in the same β-sheet. The distance in the native protein structure between the backbone H-bond donor and acceptor in L44 and V55 of the strands β1 and β3 is too large (4.69 Å) to be bridged by a single H-bond. Two H-bonds from the threonine side chain at position 54 bridge the two strands and thereby stabilize the local β-sheet conformation. T54S is a conservative substitution with a preserved hydroxyl group and identical H-bonding pattern, whereas T54A is a non-conservative mutation. The missing hydroxyl group in T54A is expected to have an impact on the H-bonding pattern and the local β-sheet conformation, possibly impacting inhibitor binding.

The same expectation holds for the conformation of the neighboring loop consisting of the residues V55, Y56, H57 and G58. T54 is located next to this loop structure (Figures 2 and 5b), which is involved in shaping the protease surface and the cavity accommodating the cyclopropyl group. Local conformational changes upon mutation at T54, particularly T54A, are expected to have an impact on the succeeding loop, affecting the cavity conformation and the residues Q41, F43 and H57 involved in direct interactions with the VX-950 cyclopropyl group (Figure 4).

We did not observe non-covalent interactions between T54 and the catalytic triad residues consisting of H57, D81 and S139. However, catalytic triad residues interact directly with residue V55, which follows T54. In addition, residues T54 and V55 interact via an H-bond (Figure 5c). Therefore, T54 interacts with each of the catalytic residues indirectly via V55. Together with the structural changes found in the ligand binding site (see 'Molecular dynamics simulations of T54 mutant structures' described below), a potential impact of the mutation T54A on catalytic residues might explain effects on the catalytic activity of the protease NS3-4A. We found no direct non-covalent interaction of T54 with G137, a residue of the oxyanion hole. Nevertheless, an indirect effect could occur via residue L44 and two edges (see network in Figure 4).

### Molecular dynamics simulations of T54 mutant structures

**Figure 6:**
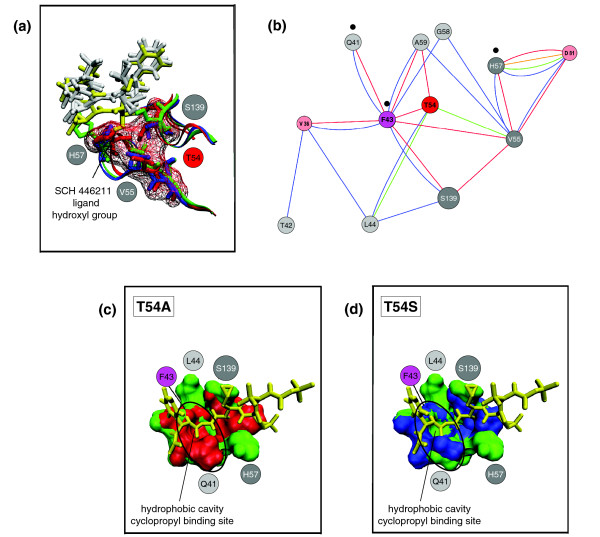
MD simulations of T54 mutants. **(a) **Three-dimensional structure analysis of the NS3-4A protease binding pocket taken from the equilibrated structures of the MD simulations of the wild-type (PDB entry 2FM2) and the T54A and T54S mutants (all with ligand SCH 446211). The wild-type protease structure is shown in green and its ligand in yellow. The mutants T54A and T54S are colored red and blue, respectively, and the corresponding ligands white. Part of the surface of the T54A mutant structure is shown in wireframe representation. For the sake of clarity, we have not included the surfaces of the wild-type protein and the T54S mutant structure. The side chains of the residues H57 and S139 in the wild-type structure extend out of the surface of the mutant structure. One of the hydroxyl groups of the SCH 446211 ligand in the wild-type structure clashes with the mutant's surface. Thus, in the mutated structures, this group is rotated by about 90 degrees to the upper right of the inhibitor. **(b) **Structural changes observed by MD simulations are reflected by the corresponding network of non-covalent residue interactions. Important conformational changes occur at L44 and V55, which do not directly interact with the ligand. Only H57 and S139 contact the ligand, and H57 forms direct interactions with the cyclopropyl group of VX-950. Nodes in the network are highlighted according to the MD simulation results (for details, see legend of Figure 5). **(c) **Protein surface of the binding pocket for the wild-type protease structure (green) with the ligand SCH 446211 (yellow) in comparison to the T54A mutant structure (red). The changes in the surface (circled in black) correspond to considerable side chain movements as discussed in (a). **(d) **Protein surface of the binding pocket for the wild-type protease structure (green) with the ligand SCH 446211 (yellow) in comparison to the T54S mutant structure (blue). The changes in the surface area (circled in black) correspond to considerable side chain movements as described in (a).

We investigated conformational changes upon mutation at T54 by molecular dynamics simulations. Simulations were performed for the wild-type structure 2FM2 and the mutants T54A and T54S (see Materials and methods). We predominantly observed two effects. First, both mutations T54A and T54S yield considerably decreased side chain volumes in comparison to the wild-type structure, leading to joint side chain rearrangements of the residues V55, H57 and S139 surrounding residue T54 (Figure 6a). The changes observed in side chain orientation are more pronounced for the T54A mutant than for the T54S mutant. During the observed rearrangements, the side chain of V55 is rotated, allowing the five-membered ring of H57 to rotate and the side chain of S139 to rotate towards the protein interior. This observation is in agreement with the results of our analysis of the residue interaction network (Figure 5c), which shows the relevance of V55 regarding the structural integrity of the catalytic site of the protease. This finding can readily be explained by the smaller size of the side chain of alanine in contrast to serine or threonine and by the reduced capability of alanine to form H-bonds. Notably, the observed relevance of the L44-T54-V55 H-bonding pattern for the impact of mutations at T54 is in good agreement with our findings from studying the residue interaction network (Figure 5a). Another observed effect is a change in the depth of the binding pocket between the wild-type structure and the T54A mutant, which possibly has an impact on protease-ligand interactions. This depth change is noticeable for the region formed by the five residues, Q41, T42, F43, G58 and A59. Residues Q41, T42 and F43 are connected to residue T54 via van der Waals interactions. F43 interacts directly with T54, whereas residues Q41 and T42 interact indirectly with it (Figure 6b). The aromatic ring of F43 is located directly next to the side chain of T54. Due to the considerable decrease in side chain volume of the T54A mutant and its hydrophobicity, this aromatic ring moves towards the protein interior of the mutant structure (Figure 6a). In addition, L44 forms an H-bond with the hydroxyl group of the side chains of both T54 and S54 (Figures 5a and 6b) and establishes van der Waals interactions with residues Q41 to F43. In contrast, this H-bond does not exist in the T54A mutant. This missing H-bond to L44 and the change in side chain orientation of F43 lead to a shallower cyclopropyl binding pocket in the T54A mutant compared to the wild-type structure; no such effect is observed for the T54S mutation.

In general, the surface and hydrophobic cavity are shallower in the mutant structure T54A than in the wild type, but this is not the case for T54S. Figure 6c illustrates the decreased volume of the cavity using the surface of the mutant structures, which covers the surface of the wild-type structure in the cyclopropyl binding pocket. In summary, the molecular dynamics simulations for T54A/S mutant structures corroborate the previous analysis of the residue interaction network. Both studies suggest a conformation change at the binding site for the T54 mutants.

### Mutations at position V36

**Figure 7:**
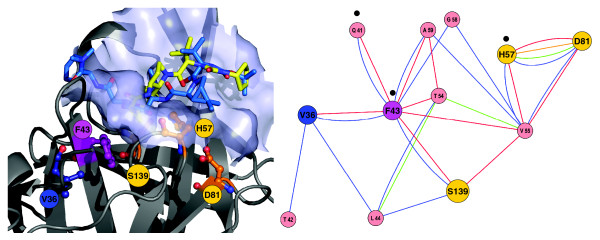
Visualization of the NS3-4A protease binding pocket (left) and the corresponding network of non-covalent residue interactions in the neighborhood of F43 (right). For details, see the legend of Figure 5.

Both in the three-dimensional structure and within the residue interaction network, V36 is more distant than T54 from the residues (Q41, F43 and H57) involved in interactions with the cyclopropyl group of VX-950 (Figure 7). In the interaction network, we identified two types of van der Waals interactions between V36 and F43, backbone-side chain and side chain-side chain. In particular, F43 is directly involved in forming the hydrophobic cavity and in interactions with the cyclopropyl group. F43 is also linked by two edges to Q41.

The network distance between V36 and the catalytic residues is larger than between T54 and the same residues. V36 interacts indirectly with S139 via a two-edge path including F43. At least three to four edges in the network need to be traversed to reach the other catalytic residues H57 or D81. No direct non-covalent interaction is present between V36 and any of the catalytic residues H57, D81 or S139. Similarly, there is no direct non-covalent interaction between V36 and the oxyanion hole at G137. An indirect interaction of V36 with G137 is possible via two edges (see network in Figure 4).

### Rotamer analysis of V36 mutations

**Figure 8:**
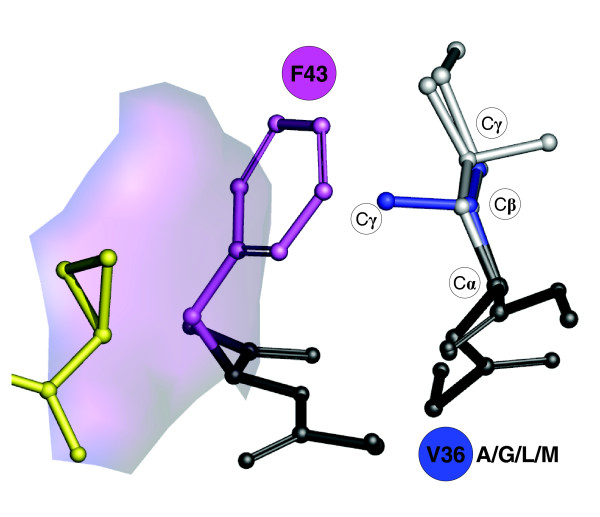
Rotameric states and conformational differences for V36 mutants A/G/L/M computed with IRECS using the PDB entry 1RTL. The figure illustrates the relative position of mutant side chains (light grey) and the wild-type residue V36 (blue). The important carbon atoms of the side chains are indicated as C_α_, C_β _and C_γ_. Protein backbone changes are depicted in black. The contribution of F43 to the hydrophobic cavity conformation and the cyclopropyl binding pocket is illustrated by means of a transparent surface patch.

We predicted side chain conformations of the mutated residues A/G/L/M at position V36 using IRECS [33]. Figure 8 illustrates potential side chain conformations for the wild-type residue V36 and the A/G/L/M mutants. Our analysis reveals that: all side chains are oriented towards the protein center and away from the ligand-binding pocket; and one C_γ _atom in the side chain of the mutant residues and a second C_γ _atom of the wild-type V36 point towards the aromatic ring of F43. The second C_γ _carbon of V36 is engaged in van der Waals interactions with the aromatic ring of F43. There is no equivalent to the second C_γ _carbon in the V36 mutants A/G/L/M. Therefore, a slight displacement of the F43 side chain towards the protein interior can be expected in the mutant structures relative to the wild-type structure. In particular, the residue interaction network in Figure 7 demonstrates that changes at F43 can impact the conformation of the catalytic residue S139 and of Q41 and its binding to the cyclopropyl group of VX-950.

### *In vitro *analysis of the V36G resistance mutation using an HCV replicon-assay

Based upon our previous analysis, we performed a comparison of antiviral efficacies for the two protease inhibitors VX-950 and SCH 503034. Only the SCH 503034 inhibitor is lacking the cyclopropyl group (Figure 1). We used a wild-type HCV replicon assay (genotype 1b) and an assay harboring the V36G mutant for *in vitro *testing. Detailed information on experimental procedures is given in Materials and methods. We found that the SCH 503034 inhibitor is efficient on the V36G mutant with effective suppression of viral RNA titers and a mean IC_50 _value clearly below 5 μM. In contrast, VX-950 was less effective in the V36G mutant replicon assay, with an IC_50 _value of about 5 μM. In comparison to the wild-type replicon assay, viral suppression was considerably delayed only for VX-950 in the V36G mutant assay. SCH 503034 was nearly equally effective in viral suppression for both the V36G mutant assay and the wild-type assay (Figure S3 in Additional data file 1).

### Comparison of HCV genotypes

**Figure 9:**
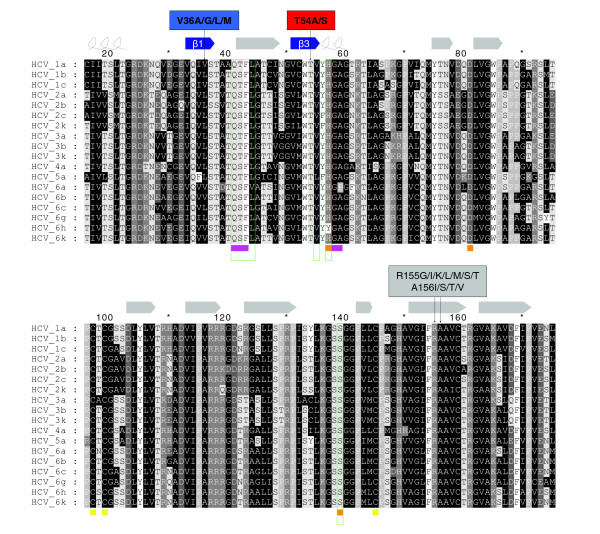
Multiple alignment of NS3 protease sequences for different HCV genotypes. UniProtKB accession numbers are given in Table S1 in Additional data file 1. The aligned sequences contain amino acids 16 to 176 according to the PDB entry 1DY8 (UniProtKB accession number P26662). The DSSP secondary structure assignment for 1DY8 is illustrated at the top of the alignment with curled lines for α-helices and arrows for β-strands. The catalytic triad consisting of H57, D81 and S139 and a zinc finger formed by C97, C99 and C145 is indicated at the bottom of the alignment by orange and yellow squares, respectively. The binding cavity for the cyclopropyl group of the VX-950 compound and CPX ligand is marked by purple squares. Text labels annotate different sites of drug resistance mutations (V36, T54, R155, A156) for VX-950. Amino acids are shaded in different grey levels according to their physicochemical properties: aliphatic (A/C/G/I/L/M/N/Q/V), black (white letters); aromatic (F/W/Y), white (black letters); cyclic (P), light grey (white letters); basic (H/K/R), grey (white letters); acidic (D/E), dark grey (white letters). Amino acids with conformational changes described in the paper are framed by green boxes.

We analyzed residues in the cyclopropyl binding cavity of the NS3-4A protease and at the mutational sites V36 and T54 with respect to their inter-genotype variability based on the recent HCV genotype nomenclature [34]. The cavity-forming residues Q41, T42, F43, H57, G58 and A59 are strongly conserved throughout the investigated HCV sequences. We observed a conservative T42S polymorphism in about 61% of the sequences. The only non-conservative polymorphisms H57Y and A59P are found in genotypes 6h and 6a, respectively. These results point to overall similar shapes and physicochemical properties of the cavities in NS3 protease domain structures, resulting in comparable binding modes of the VX-950 cyclopropyl group for all HCV genotypes investigated. Regarding the mutational sites V36 and T54, we found a conservative V36L polymorphism in about 67% of all sequences, with L36 in genotypes 1b, 2, 3, 4, 5 and 6c/g. In contrast, T54 is strictly conserved in all HCV genotypes (Figure 9). Considering our rotamer analysis and the importance of the number of side chain C_γ _atoms at position 36 for the F43 side chain conformation, we can conclude that HCV sequences with L36 (with only one C_γ _atom) should be less susceptible to drug resistance mutations at this site, especially the clinically more relevant HCV genotypes 1b, 2 and 3. F43 seems to be important for resistance development at V36 and T54 and has a conservation of 100%. L44 was found to be involved in mechanisms of drug resistance at T54 and showed a conservation of 94%. A conservative L44V polymorphism was found in HCV genotype 6a. Position 55, which was supposed to be responsible for impaired catalytic activity in T54 protease mutants, was conserved in 94%. A conservative V55L polymorphism was found in HCV genotype 5a.

## Discussion

Our results indicate that the cyclopropyl group of VX-950 is oriented towards a hydrophobic cavity in the binding pocket of the HCV protease NS3-4A. The cyclopropyl binding mode and the geometry of the cavity appear to play an essential role in the development of drug resistance by mutants at positions V36 and T54. The residue T54 lies in an anti-parallel β-sheet, which is followed by a loop structure involved in shaping the hydrophobic cavity. We expect a larger impact of T54A than T54S on the β-sheet conformation due to the affected H-bond formation.

Molecular dynamics simulations of T54A/S mutant structures support our interpretation. We observed more pronounced structural changes in the case of T54A compared to T54S, which impact the binding pocket, particularly at the hydrophobic cavity that accommodates the cyclopropyl group. We also observed a reduced depth of the cyclopropyl binding cavity for the T54A mutant structure. *In vitro *data for T54A revealed an 11.7-fold increase of IC_50_, whereas T54S showed only a minimal level of drug resistance, with a 1.9-fold increase in IC_50 _(Table 1) [19,26-28]. We suppose that the minor impact on the protease structure and the less compromised VX-950 binding in the case of T54S results in low-level drug resistance, in contrast to T54A with higher drug resistance levels. Furthermore, we analyzed potential molecular mechanisms affecting catalytic residues of the NS3-4A protease and the implications for viral replication efficacy. A network of non-covalent residue interactions demonstrated possible effects of T54 mutants not only on the ligand binding site, but also on the catalytic residues. This is in agreement with results of molecular dynamics simulations upon T54A/S mutation and underlines the considerable negative influence of T54 mutants on the protease catalytic activity.

We found V36 to be located farther away from the hydrophobic cavity than T54, both in the three-dimensional structure and in the residue interaction network derived from the NS3 protease structure. We observed non-covalent interactions of the wild-type V36 with a residue that shapes the hydrophobic cavity. The mutations V36A/G/L/M allow a displacement of the side chain of this residue, thereby changing the shape of the cavity. Thus, the V36 mutants affect only the shape of the cyclopropyl binding cavity, which is in agreement with the corresponding low-level drug resistance and weak IC_50 _fold changes of only 1.7 to 6.9 (Table 1) for V36A/L/M single mutations [19,26-28]. We conjecture that the binding affinities of the VX-950 compound are modified only marginally, which is consistent with the low-level drug resistance. The residue V36 and its mutants are only of minor relevance for the protease catalytic activity. In comparison with T54, we observed lower network connectivity and larger distance from catalytic triad residues in the network for the V36 node. This may explain why V36 mutants have been observed in all breakthrough patients and more frequently in follow-up sequencing data than T54 mutants, which indicates greater protease enzymatic activities and better viral replication efficacies [18,19,26-28]. After withdrawal of VX-950, V36 mutants remained at a fairly steady frequency in HCV quasispecies populations, most probably due to an only slightly decreased viral replication rate and a low-level drug resistance [18,19,26-28].

Moreover, we performed a comprehensive comparison of NS3 protease sequences for all HCV genotypes. We found only minor variability at the mutational sites and residue positions investigated in this study. The clinically most relevant HCV genotypes 1, 2 and 3 are particularly similar in contrast to other genotypes. Altogether, we assume closely related molecular resistance mechanisms for all HCV genotypes when treated with VX-950 or compounds with a similar scaffold.

## Conclusion

We identified a narrow hydrophobic cavity in the binding pocket of the protease NS3-4A accommodating the cyclopropyl group of VX-950 (telaprevir). Mutations at V36 and T54 are expected to affect local conformation and the geometry of this cavity, which explains the observed drug resistance. We used a structural network of non-covalent interactions between NS3 protease residues to investigate molecular effects underlying drug resistance. Notably, this novel methodological approach is of general applicability for many studies of protein structure and function. In our work, the residue interaction network allowed the identification of key mechanisms responsible for conformational changes in the ligand binding pocket and hydrophobic cavity as well as for functional effects on the protease catalytic residues. Molecular dynamics simulations and rotamer analysis support our findings well. Additionally, we performed experimental inhibitor studies with VX-950 and SCH 503034 in a mutant HCV replicon assay, which corroborated our results.

Based on the present work, we conclude that add-on or switch to complementary protease inhibitors, possessing no cyclopropyl or similar group in an equivalent position as in VX-950, might help to avoid cross-resistance during viral breakthrough and follow-up. Therefore, we suggest further experiments to examine our observations. NS3 protease mutants could be tested for their antiviral efficacy and compromised viral replication. Based upon our findings, it would be of interest to compare the efficacy of VX-950 against that of SCH 503034 for other V36 and T54 mutants. Apart from that, crystal structure information would be desirable for mutant structures with co-complexed drugs like VX-950 to confirm our computational analysis.

## Materials and methods

### Analysis of experimental structural models of NS3-4A

Alternative experimental structure models of the HCV protease NS3 were compared based on the differences of the intramolecular distances using the backbone carbon alpha (C_α_) atoms and the geometric centers of the side chain atoms [35]. In total, 37 experimental models available in the PDB [13,14] were analyzed, including five structure models lacking NS4A. The 32 different structure models of the NS3-4A protease were superimposed for further analysis, excluding the five models without NS4A due to major conformational differences. Invariant structural regions were identified and superimposed [35]. Multiple structure models determined by X-ray crystallography are normally available from each PDB entry because it includes more than one protease domain in the asymmetric unit. We used PyMOL [36] for the visualization of protein structure images. Chimera [37] was used for the calculation of buried cavities within the NS3-4A protease (PDB entry 2FM2) and the derived mutant structures.

### Protein-ligand docking

The protein-ligand docking of VX-950 was performed using PDB entry 1RTL of the protease NS3-4A. The binding pocket was defined as a subset of all residues that have at least one atom closer than 6.5 Å to any atom of the 1RTL ligand. The ScreenScore [38] parameterization of the docking program FlexX [39] was applied to account for the mainly hydrophobic nature of VX-950 and to compensate small-scale induced-fit effects because ScreenScore uses a softer consensus scoring function than the standard FlexX does. The chemical structure of the ligand VX-950 (Figure 1) was drawn with MDL ISIS/Draw [40]. The three-dimensional structure was derived by energy minimization with MMFF94. The cyclopropyl group of VX-950 was selected as the base fragment of FlexX to achieve a high sampling rate on this group. First, VX-950 was docked into the binding pocket without specifying a covalent bond. FlexX automatically places VX-950 in a non-covalent binding mode so that the cyclopropyl group is placed in the same hydrophobic region as the CPX ligand in 1RTL and so the ketone oxygen is nearby the S139 side chain. Next, we fixed the covalent bond and relaxed the structure of VX-950 using a 100-step energy minimization. We chose this two-step setup to ensure that the docking is not biased by the geometrical constraints of a predefined covalent bond. The covalent bonding was observed for analogous ketoamide inhibitors [25] and ketoacid inhibitors [12]. We used MOE (Molecular Operating Environment) [41] to visualize ligand interaction diagrams for 1RTL and 2FM2 ligands and PyMOL [36] for the visualization of the VX-950 docking results.

### Network of non-covalent interactions

In the following, the term interaction denotes non-covalent interactions. The non-covalent H-bond and van der Waals interactions between amino acids were identified in PDB entry 1RTL and represented as a two-dimensional network. We used the WHAT IF web interface [42,43] to identify H-bonds and van der Waals interactions between residues. The network was visualized in Cytoscape [44]. LIGPLOT [45] was additionally applied to identify H-bonds and van der Waals interactions of the ligand with amino acids of the protease NS3-4A. The local connectivity of each residue was calculated as the number of its interactions using the Cytoscape plugin NetworkAnalyzer [46,47], which suggested residues of functional importance in the interaction network. Distances between residue nodes were computed by NetworkAnalyzer as the minimum number of interaction edges connecting two nodes.

### Rotamer analysis and side chain orientation

We predicted side chain conformations of the mutated residues V36A/G/L/M with the tool IRECS (Iterated Restriction of Conformational Space) [33]. IRECS analyses ensembles of possible rotameric states of side chains and subsequently filters out states with unlikely interaction patterns with the backbone and other side chains.

### Molecular dynamics simulations

Molecular dynamics (MD) simulations were performed for the wild-type structure of the NS3-4A protease and the mutants T54A and T54S using the program GROMACS3.3 [48]. Regarding the mutant structures, the wild-type structure (PDB entry 2FM2) was used and the corresponding side chain (T54) was mutated using the tool IRECS [33]. The original ligand SCH 446211 from 2FM2 was used for the simulations. This choice was based on the fact that the goal of the simulations was to evaluate the influence of the mutations on the protein structure in general, which should be the same regardless of the bound ligand and should not depend on the binding of a specific ligand. Thus, we used the original experimental ligand SCH 446211 in order to avoid potential artifacts originating from docking inaccuracies in our simulation. The analysis of the simulation results was based on the final structures of the simulations after equilibration. For the simulations, the GROMOS96 force field [49] and the SPC water model were used, applying periodic boundary conditions. The long-range non-bonded interactions were treated by particle-mesh Ewald summation, and a time step of 2 fs was used. Throughout the simulations, the bond lengths were constrained to ideal values using the LINCS procedure [50]. The system was heated from 0 to 300 K over 120 ps, and the simulations were then continued at 300 K and at a constant pressure of 1 atm for 2 ns. The temperature and pressure were maintained by weak coupling to an external bath with a temperature coupling relaxation time of 0.1 ps and a compressibility of 4.5·10^-5 ^[51]. For the analysis of MD simulation results, the average structures of the protease-inhibitor complexes of the last 100 ps of the simulations were used, superimposing backbone C_α _atoms. On the basis of these structures, the conformations of the residues in the binding pocket and the pocket surface were analyzed. The tool VMD [52] was applied for the visualization of simulated protease-ligand structures.

### Multiple sequence analysis

Sequences of the different HCV variants of the NS3-4A protease were retrieved from the UniProtKB database [53,54]. HCV genotypes are named according to a recent consensus proposal for a unified system of HCV genotype nomenclature [34]. The UniProtKB accession numbers of the sequences reported in this paper are given in Table S1 in Additional data file 1. A multiple sequence alignment (Figure 9) of the NS3-4A protease domain was computed using MUSCLE [55] and subsequently improved by minor manual modifications using the SEAVIEW alignment editor [56]. The secondary structure assignment to the PDB structure 1DY8 was taken from the DSSP database [57,58]. The sequence alignment figure was illustrated using GeneDoc [59]. Residue numbering in the manuscript is according to HCV genotype 1b, PDB entry 1DY8.

### *In vitro *IC_50 _determination of mutant NS3-4A proteases

#### Compounds

VX-950 was synthesized by the European Network of Excellence for Viral Resistance in Hepatitis C (viRgil, Drugpharm), dissolved in dimethyl sulfoxide as a 6.6 mM solution. SCH 503034 was synthesized by Schering-Plough Corporation (Kenilworth, NJ, USA), dissolved in dimethyl sulfoxide as a 19 mM solution. Both compounds were stored at 4°C.

#### Plasmids and *in vitro *RNA transcription

The plasmid pFKI_389_neo/NS3-3'/ET contains HCV subgenomic replicon sequences derived from HCV genotype 1b and an upstream T7 promoter for *in vitro *RNA synthesis. The point mutation was generated with the QuikChange II XL Site-Directed Mutagenesis Kit (Stratagene, La Jolla, CA, USA). The plasmid was linearized with *Sca*I and purified by phenol chloroform extraction. The linearized and purified plasmid was transcribed by using a T7 RNA polymerase (Promega, Madison, WI, USA) according to the manufacturer's instructions. All of the plasmids and RNAs were checked for purity and integrity by standard procedures.

#### Generation of HCV replicon cell lines

Huh-7.5 cells were cultured in Dulbecco's modified Eagle's medium (DMEM; Invitrogen, Carlsbad, CA, USA) containing 10% fetal bovine serum (FBS; PAA Laboratories GmbH, Pasching, Germany) and 2 mM L-glutamine. The cells were transfected with an *in vitro*-transcribed subgenomic HCV replicon RNA. The wild-type sequence was identical to that of the pFKI_389_neo/NS3-3'/ET replicon [60]. Stable cells containing the self-replicating HCV replicon were selected and maintained in the presence of 750 μg of G418 (Invitrogen) per ml and were used for HCV replicon assays.

#### Two-day HCV replicon assay

HCV replicon cells were plated in a 6-well plate at a density of 2 × 10^5 ^cells per well in DMEM with 10% FBS. On the following day (24 h later), the culture medium was replaced with DMEM containing either no compound as a control or compounds serially diluted in the presence of 10% FBS and 750 μg/ml G418. After the cells were incubated with the compounds for 48 h, the intracellular RNA was extracted with an RNeasy kit (Qiagen, Valencia, CA, USA). The level of HCV RNA was determined by a real-time quantitative reverse transcription-PCR (RT-PCR) assay (Taqman) with a pair of HCV-specific primers (5'-ACG CAG AAA GCG TCT AGC CAT-3' and 5'-TAC TCA CCG GTT CCG CAG A-3'), an HCV-specific probe (5'-6FAM-TCC TGG AGG CTG CAC GAC ACT CA XT-PH-3), and an ABI Prism 7000 sequence detection system (Applied Biosystems, Foster City, CA, USA). The IC_50 _was defined as the concentration of compound at which the HCV RNA level in the replicon cells was reduced by 50%.

## Abbreviations

DMEM, Dulbecco's modified Eagle's medium; FBS, fetal bovine serum; H-bond, hydrogen bond; HCV, hepatitis C virus; IC_50_, inhibitory concentration 50%; MD, molecular dynamics; PDB, Protein Data Bank; RT-PCR, reverse transcription-PCR.

## Authors' contributions

CW and FSD conceived, designed and performed the analysis. SS performed *in vitro *experiments. IA performed MD analysis and ligand docking. CH performed rotamer analysis and ligand docking. GM and AS performed analysis of residue interaction networks. CS conceived experiments. MA, SZ and TL conceived and designed the analysis. All authors contributed to writing the manuscript. CW, FSD, MA, SZ and TL contributed to the conceptualization of the performed analyses.

## Additional data files

The following additional data are available with the online version of this paper. Additional data file 1 includes supplementary figures and table. Figure S1 illustrates NS3-4A protease-ligand interactions. Figure S2 shows the complete network of non-covalent, H-bond and van der Waals interactions of the NS3-4A protease for the PDB entry 1RTL. Figure S3 gives results of SCH 503034 and VX-950 inhibitor studies using an HCV V36G mutant replicon assay. Table S1 lists HCV genotypes included into the multiple sequence alignment of Figure 9.

## Supplementary Material

Additional data file 1 Figure S1 illustrates NS3-4A protease-ligand interactions. Figure S2 shows the complete network of non-covalent, H-bond and van der Waals, interactions of the NS3-4A protease for the PDB entry 1RTL. Figure S3 gives results of SCH 503034 and VX-950 inhibitor studies using an HCV V36G mutant replicon assay. Table S1 lists HCV genotypes included into the multiple sequence alignment of Figure 9. Click here for file
