# Supplementary material for: Molecular basis of telaprevir resistance due to V36 and T54 mutations in the NS3-4A protease of the hepatitis C virus
Source: Genome Biol. 2008 Jan 23;9(1):R16. doi: 10.1186/gb-2008-9-1-r16 (PMC2395260; doi:10.1186/gb-2008-9-1-r16)
Supplement: Additional data file 1 — Figure S1 illustrates NS3-4A protease-ligand interactions. Figure S2 shows the complete network of non-covalent, H-bond and van der Waals, interactions of the NS3-4A protease for the PDB entry 1RTL. Figure S3 gives results of SCH 503034 and VX-950 inhibitor studies using an HCV V36G mutant replicon assay. Table S1 lists HCV genotypes included into the multiple sequence alignment of Figure 9. [file gb-2008-9-1-r16-S1.pdf]

Additional Data File 1

Molecular basis of telaprevir resistance due to V36 and T54 mutations in the NS3-4A protease of HCV

Christoph Welsch, Francisco S. Domingues, Simone Susser, Iris Antes, Christoph Hartmann, Gabriele Mayr, Andreas Schlicker, Christoph Sarrazin, Mario Albrecht, Stefan Zeuzem and Thomas Lengauer

Supplemental Figure Legends

Figure S1. NS3-4A protease-ligand interactions. **Left column:** Binding pocket surface of the NS3-4A protease for the PDB structures 1RTL **(A)** and 2FM2 **(B)**. The ligands CPX and SCH 446211 are colored in yellow and light blue, respectively. The surface of the protein was colored with the vacuum electrostatics function of PyMOL. Charges are computed with the Amber 99 force field and projected on the protein surface, whereas colored patches (red for positive, blue for negative) denote polar regions and white patches apolar protein regions. **Right column:** Corresponding MOE diagrams for protease-ligand interactions with the legend at the bottom. Additional H-bond interactions from LIGPLOT, not given by MOE, are indicated by red dotted lines.

Figure S2. Complete network of non-covalent residue interactions for the NS3-4A protease (PDB entry 1RTL). Nodes represent residues and colored edges represent different types of interactions: **(i)** van der Waals interactions: backbone – side chain (blue), side chain – side chain (red) **(ii)** H-bond interactions: backbone – side chain (green), side chain – side chain (orange). Nodes in the focus of the present analysis are colored in green.

Figure S3 In-vitro IC_{50} determination of a V36G mutant NS3-4A protease. **Left column:** Dose-dependent reduction of HCV RNA in the replicon cells with (V36G) and without (wt) mutation by SCH 503034. The replicon cells were incubated with various concentrations of SCH 503034 for 48h. At the end of the incubation period, total RNA was extracted, and the levels of HCV RNA remaining were determined by the QRT-PCR assay. These levels are shown as percentages relative to the levels of HCV RNA in cells incubated with no compound. Each data point represents the average for two cell culture replicates. **Right column:** Experimental procedures as described in the left column for VX-950.

Figure S1.

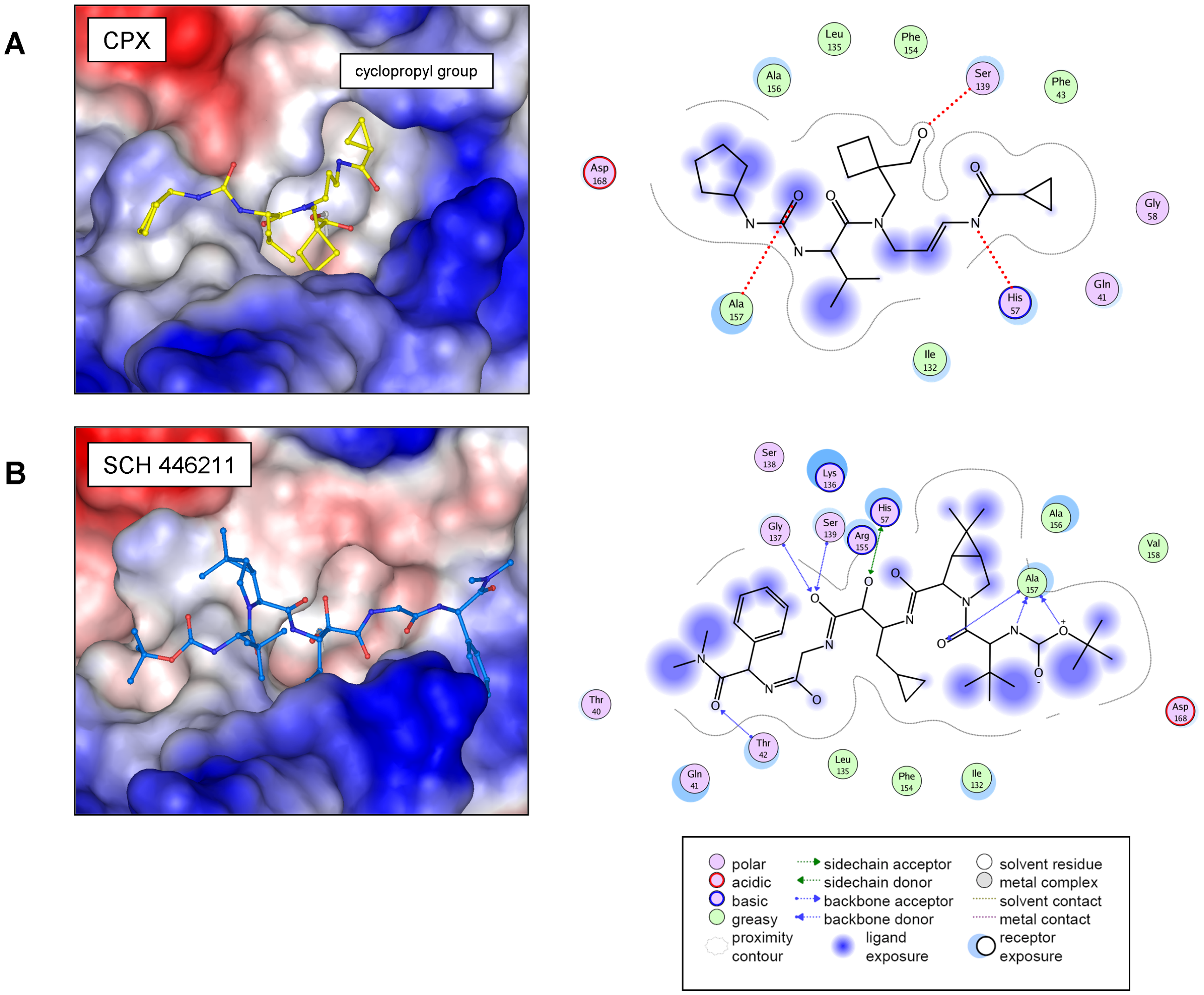
A
CPX
cyclopropyl group
B
SCH 446211
Leu 135
Phe 154
Ala 156
Ser 139
Phe 43
Asp 168
Gly 58
Gln 41
His 57
Ala 157
Ile 132
Ser 138
Lys 136
His 57
Gly 137
Ser 139
Arg 155
Ala 156
Val 158
Ala 157
Thr 40
Thr 42
Gln 41
Leu 135
Phe 154
Ile 132
Asp 168
polar
acidic
basic
greasy
proximity contour
sidechain acceptor
sidechain donor
backbone acceptor
backbone donor
ligand exposure
solvent residue
metal complex
solvent contact
metal contact
receptor exposure

Figure S2.

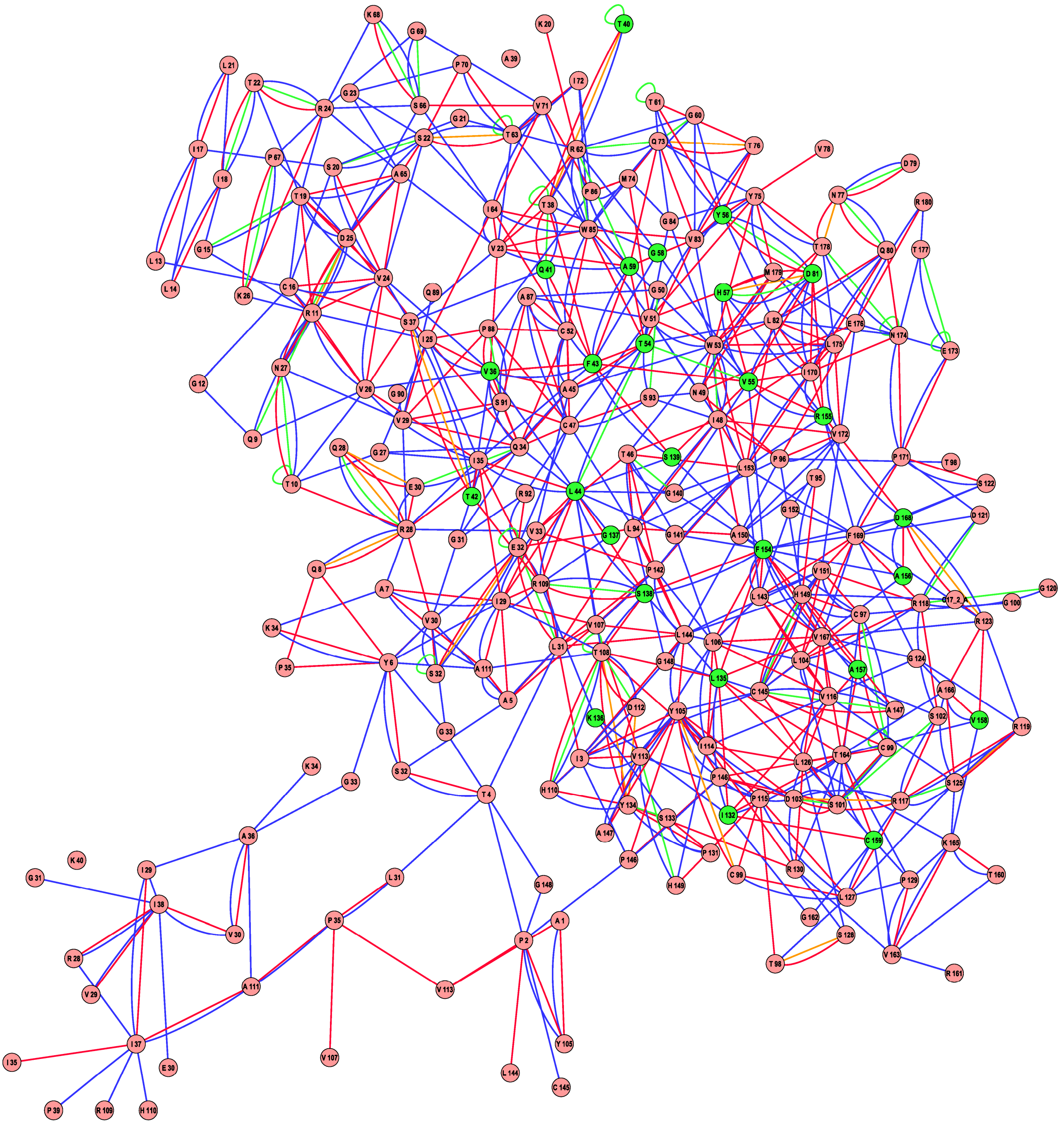

Figure S3.

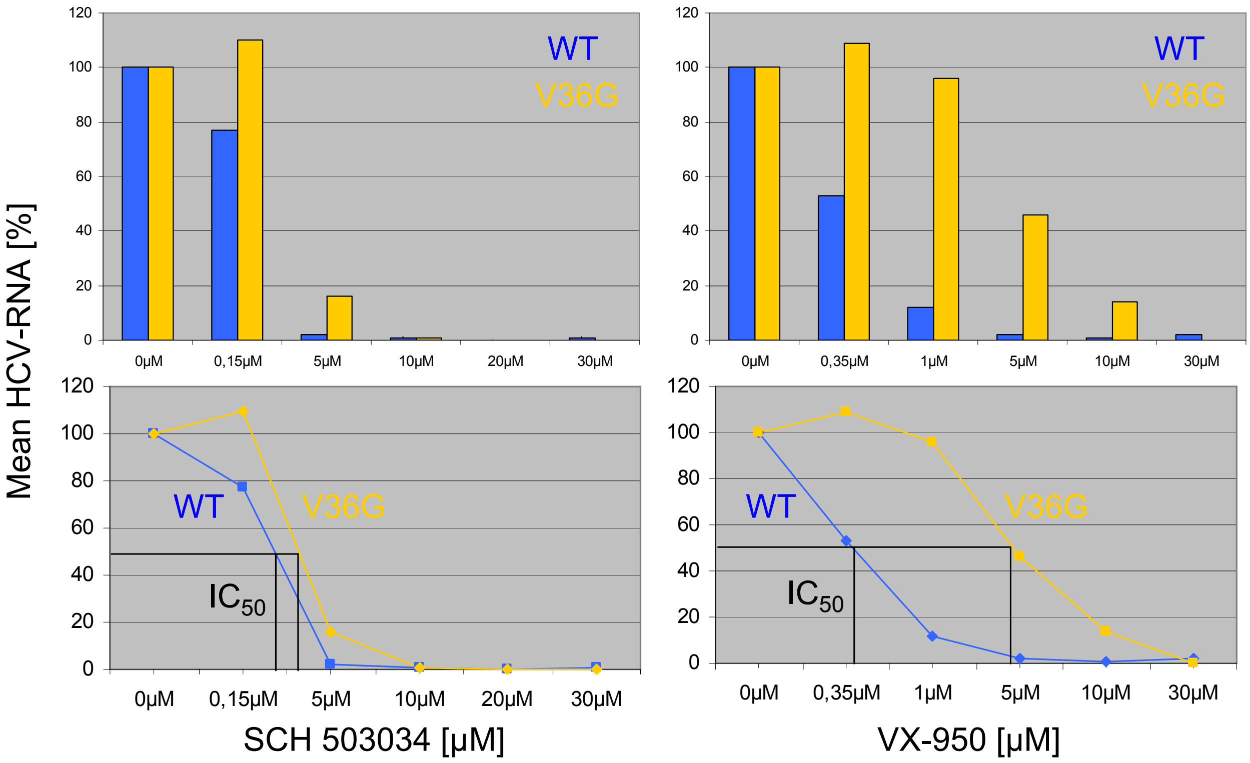
Mean HCV-RNA [%]
WT
V36G
0µM
0,15µM
5µM
10µM
20µM
30µM
0,35µM
1µM
IC50
SCH 503034 [µM]
VX-950 [µM]

Supplemental Table

Table S1. HCV genotypes included into the multiple sequence alignment in Figure 9.

UniProtKB entry name	***UniProtKB number***	***PDB structure***	***HCV genotype***	***Isolate***
POLG_HCV1	P26664	None	**1a**	1
POLG_HCVJA	P26662	1DY8	**1b**	Japanese
POLG_HCVH9	Q81754	None	**1c**	HC-G9
POLG_HCVJ6	P26660	None	**2a**	HC-J6
POLG_HCVJP	Q9DHD6	None	**2b**	JPUT971017
POLG_HCVBB	Q68749	None	**2c**	BEBE1
POLG_HCVVA	Q9QAX1	None	**2k**	VAT96
POLG_HCVNZ	Q81258	None	**3a**	NZL1
POLG_HCVTR	Q81487	None	**3b**	Tr-Kj
POLG_HCVJK	Q68801	None	**3k**	JK049
POLG_HCVED	O39929	None	**4a**	ED43
POLG_HCVEV	O39928	None	**5a**	EUH1480
POLG_HCVEU	O39927	None	**6a**	EUHK2
POLG_HCVT5	O92529	None	**6b**	Th580
POLG_HCVVN	O92530	None	**6c**	VN235
POLG_HCVJL	Q68798	None	**6g**	JK046
POLG_HCVVP	O92532	None	**6h**	VN004
POLG_HCVVO	O92531	None	**6k**	VN405
